# Childhood attention-deficit hyperactivity disorder problems and mid-life cardiovascular risk: prospective population cohort study

**DOI:** 10.1192/bjp.2023.90

**Published:** 2023-10

**Authors:** Ajay K. Thapar, Lucy Riglin, Rachel Blakey, Stephan Collishaw, George Davey Smith, Evie Stergiakouli, Kate Tilling, Anita Thapar

**Affiliations:** Division of Psychological Medicine and Clinical Neurosciences, Centre for Neuropsychiatric Genetics and Genomics, Wolfson Centre for Young People's Mental Health, Cardiff University School of Medicine, Cardiff, UK; Population Health Sciences and MRC Integrative Epidemiology Unit, University of Bristol, Bristol, UK

**Keywords:** Childhood, ADHD, mid-life cardiovascular risk, cohort study, epidemiology

## Abstract

**Background:**

It is well-known that childhood attention-deficit hyperactivity disorder (ADHD) is associated with later adverse mental health and social outcomes. Patient-based studies suggest that ADHD may be associated with later cardiovascular disease (CVD) but the focus of preventive interventions is unclear. It is unknown whether ADHD leads to established cardiovascular risk factors because so few cohort studies measure ADHD and also follow up to an age where CVD risk is evident.

**Aims:**

To examine associations between childhood ADHD problems and directly measured CVD risk factors at ages 44/45 years in a UK population-based cohort study (National Child Development Study) of individuals born in 1958.

**Method:**

Childhood ADHD problems were defined by elevated ratings on both the parent Rutter A scale and a teacher-rated questionnaire at age 7 years. Outcomes were known cardiovascular risk factors (blood pressure, lipid measurements, body mass index and smoking) at the age 44/45 biomedical assessment.

**Results:**

Of the 8016 individuals assessed both during childhood and at the biomedical assessment 3.0% were categorised as having childhood ADHD problems. ADHD problems were associated with higher body mass index (*B* = 0.92 kg/m^2^, s.d. = 0.27–1.56), systolic (3.5 mmHg, s.d. = 1.4–5.6) and diastolic (2.2 mmHg, s.d. = 0.8–3.6) blood pressure, triglyceride levels (0.24 mol/l, s.d. = 0.02–0.46) and being a current smoker (odds ratio OR = 1.6, s.d. = 1.2–2.1) but not with LDL cholesterol.

**Conclusions:**

Childhood ADHD problems predicted multiple cardiovascular risk factors by mid-life. These findings, when taken together with previously observed associations with cardiovascular disease in registries, suggest that individuals with ADHD could benefit from cardiovascular risk monitoring, given these risk factors are modifiable with timely intervention.

Attention-deficit hyperactivity disorder (ADHD) is a common neurodevelopmental condition with an estimated prevalence in children and adolescents of 3–5%.^1^ It typically first manifests during childhood, although most individuals continue to have symptoms and impairment in adolescence and adult life.^2^ As there are multiple barriers to accessing specialist healthcare, even in high-income countries, many people with ADHD fail to receive a diagnosis and appropriate treatment. This is despite evidence that ADHD is associated with multiple adverse mental health, educational and social outcomes.^3–5^ More recently there is growing evidence that ADHD may be a risk factor for physical ill health and premature mortality.^6^ Recent studies of patient populations including large Scandinavian registry-based studies have confirmed risks between clinically recognised ADHD and a diagnosis of cardiovascular disease (CVD).^6^ A two-sample Mendelian randomisation approach also found that genetic liability for ADHD may be causal for coronary artery disease.^7^ As ADHD is known to be associated with higher rates of cigarette smoking and elevated body mass index (BMI), these risk exposures could explain elevated rates of CVD in people with ADHD. However, observed associations could also be explained by additional CVD risk factors.

## Cardiovascular risk factors

Major CVD risk factors include elevated blood lipids (particularly low-density lipoprotein (LDL) cholesterol and triglycerides), elevated blood pressure, cigarette smoking and obesity/elevated body mass index (BMI), as well as genetic liability.^8^ CVD can be prevented by targeting many of these risk factors (e.g. hypertension, high lipids) for intervention and CVD risk prediction tools have therefore been adopted in primary care settings in many countries but not in psychiatry clinics.

## The need for population-based studies

Although clinical studies and registry data are invaluable, detailed data on lifestyle and cardiovascular risks are not routinely available. Also, given known referral biases, all clinical studies are subject to observed associations arising from ascertainment and collider bias, especially as many people with ADHD are never clinically recognised. Another challenge is that findings, particularly for systolic blood pressure, may be confounded owing to widespread use, in recent clinical cohorts, of ADHD medications known to have cardiovascular side-effects.^7^ Prospective population-based studies have different strengths compared with clinical studies. However, studies to date that have examined links between ADHD and physical health have been cross-sectional or only examined outcomes into early adult life. To the best of our knowledge, there are no prospective population-based studies that span birth to mid-life and that have allowed examination of links between childhood ADHD problems and CVD risk factors in mid-life. We used prospective data from a UK birth cohort, the National Child Development Study, in which participants were recruited at birth in 1958.^9^ This study collected information on childhood ADHD symptoms using the most widely validated measure at that time and followed up individuals into mid-life (aged 44–45 years), when biomedical data were collected. As ADHD behaves as a risk dimension, broader definitions of ADHD show associations with the same risk factors, correlates and outcomes as an interview-derived DSM-5/DSM-IV or ICD-10 diagnosis.^10^ Our aim was to examine whether ADHD problems in childhood were associated with specified CVD risk factors at age 44.

## Method

### Sample

The National Child Development Study (NCDS) is a prospective UK birth cohort study comprising 18 558 children born in England, Wales and Scotland on 3–9 March 1958. A research nurse visited participants aged 44–45 years for a face-to-face biomedical assessment, which included a detailed assessment of their physical health.

The authors assert that all procedures contributing to this work comply with the ethical standards of the relevant national and institutional committees on human experimentation and with the Helsinki Declaration of 1975, as revised in 2008. All procedures involving human subjects/patients were approved by South East Medical Research and Ethics Committee (NCDS Biomedical, Age 44, 2002, REC number 01/1/44). A comprehensive description of the NCDS study has been published.^9^ Informed parental consent was sought for the childhood measures and explicit written consent was obtained for the biomedical assessment.^11^

### Measures

#### Exposure variable: ADHD

When NCDS participants were aged 7, their mothers completed the (modified) Rutter A scale, which is a questionnaire measure of various child mental health problems, including ADHD, developed by Michael Rutter in the 1960s. The ADHD items have been validated against interview-assessed research diagnoses of ADHD.^12^ The NCDS Rutter Scale includes items on whether the child ‘is squirmy or fidgety’ or ‘has difficulty in settling’. Response categories were: ‘never’, ‘sometimes’ and ‘frequently’.^13^ Teacher reports on these two ADHD symptoms in the participants (‘squirmy, fidgety’ and ‘hardly ever still’) were also obtained at similar time points and derived from the Bristol Social Adjustment Guides (BSAG),^13^ with response categories ‘don't know’, ‘certainly applies’, ‘applies somewhat’ and ‘does not apply’. No measures of ADHD have been included in NCDS after childhood/adolescence.

Individuals were classified as having ‘ADHD problems’ if the parent endorsed both ADHD symptoms (regardless of frequency) and the teacher endorsed either of the ADHD symptoms as ‘certainly applies’, as a clinical diagnosis of ADHD requires difficulties across different settings. This approach generated a prevalence rate of 3% for ADHD problems, which is in line with global prevalence rates of DSM-IV/DSM-5 ADHD.^1^ Time trends research suggests that prevalence rates for ADHD have not changed over time.^14^

#### Outcomes: CVD risk factors assessed in ‘mid-life’ (age 44–45)

For the purposes of these analyses, all outcomes were defined as continuous variables (apart from smoking status). To aid clinical interpretation, the four categorical outcomes using current UK guidelines for clinical action (e.g. hypertension, hyperlipidaemia) were also generated:

- Blood pressure (mmHg): three readings of systolic (SBP) and three readings of diastolic (DBP) blood pressure were available. A mean value for each (SBP and DBP) was derived. Given previous evidence,^8,15^ systolic blood pressure was considered as the primary outcome.
- Lipid measurements (mmol/L): low-density lipoprotein (LDL) and triglyceride levels.
- Body mass index (BMI): height and weight were measured by nurses during home visits and BMI derived using the standard formula (weight/height, kg/m^2^). The principal measure of BMI was mid-life BMI, but we also checked whether substituting BMI at age 16 for mid-life BMI influenced the results.^16^
- Smoking status: this was based on self-report using a derived dichotomised item (‘Current smoker’, yes/no). Smoking is a well-established risk factor for CVD but also known to be strongly associated with ADHD.

### Analysis

There were three main steps in the analyses.

#### Mid-life cardiovascular risk factors as outcome

Regression analyses were conducted with childhood ADHD as exposure and cardiovascular risk factors (SBP, DBP, BMI, current smoking status, triglycerides and LDL) as separate outcomes.

#### Sensitivity analyses

As there is a well-recognised effect of sex (male/female) on systolic blood pressure and as ADHD is more prevalent in males, we added sex and an interaction term between sex and ADHD to the regression analysis of ADHD group on systolic blood pressure. We conducted similar sensitivity checks for birth socioeconomic status (SES) as a covariate and interaction term. SES at birth was classified using the Registrar General's classification of the father's social class, i.e. the social class the NCDS participant was ‘born into’. The categories were condensed into a dichotomised variable ‘higher SES’ (classes I, II and III non-manual /manual) and ‘lower SES’ (classes IV, V and unclassified).

#### Missing data analysis: multiple imputation

Full descriptions of the process and results are provided in the Supplementary materials, available at https://dx.doi.org/10.1192/bjp.2023.90. We scored missing outcome measures (adult BMI, SBP, DBP, LDL cholesterol, current smoking status and triglycerides) for our analysis sample. All baseline data and outcomes reported in the main paper are unimputed. We performed multiple imputation using a multivariate normality assumption in STATA version 16.1 for Windows. We used: (a) variables in line with published recommendations for imputing missing data in the biomedical survey in the NCDS^17^ (if they predicted missingness for all/most key mid-life outcomes); (b) earlier values of primary outcomes; (c) other key mid-life variables. A full list of variables used in the imputation is given in the Supplementary materials.

## Results

**Fig. 1:**
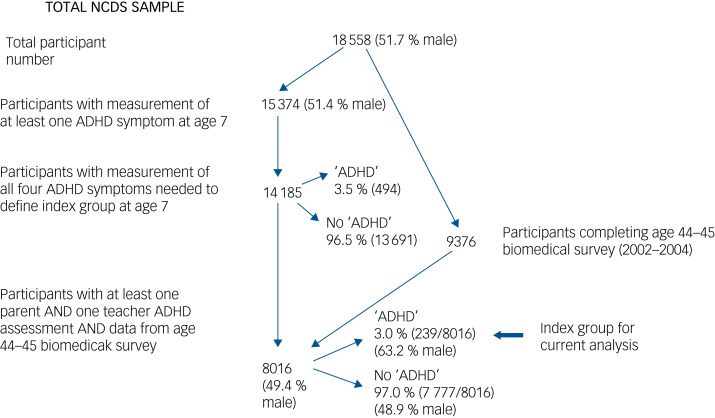
Flowchart of participant numbers. NCDS, National Child Development Study; ADHD, attention-deficit hyperactivity disorder. ‘ADHD’ indicates the presence of childhood ADHD problems (defined using two symptoms: ‘squirmy, fidgety’ and ‘hardly ever still’), rather than clinical diagnosis of ADHD (the reference to all four symptoms is for both the parent and the teacher reports).

Figure 1 is a flowchart of how the sample of participants included in our analysis was derived.

### Associations between childhood ADHD and mid-life cardiovascular risk factors

The values of mid-life cardiovascular risk factors for individuals with and without childhood ADHD problems and associations between childhood ADHD problems and mid-life cardiovascular risk factors are detailed in Table 1.

**Table 1:** Childhood attention-deficit hyperactivity disorder (ADHD) and individual mid-life cardiovascular risk factors: descriptive details and comparisons with non-ADHD group

Outcome	Exposure	Mean (s.d., *n*)	*F* (degrees of freedom)	Mean 95% CI		*P*	Effect size, *d*
				Upper	Lower		
Mid-life BMI, kg/m^2^	ADHD	28.3 (5.7), 234	7.8 (1,7871)	27.5	29.0	0.005	0.20
	Non-ADHD	27.3 (5.0), 7639		27.2	27.5		
SBP, mmHg	ADHD	129.9 (17.4), 233	10.3 (1,7892)	127.7	132.2	0.001	0.21
	Non-ADHD	126.4 (16.3), 7661		126.1	126.8		
DBP, mmHg	ADHD	80.9 (10.7), 233	9.7 (1,7892)	79.5	82.3	0.002	0.21
	Non-ADHD	78.7 (10.7), 7661		78.4	78.9		
LDL, mmol/L	ADHD	3.5 (0.97), 184	0.45 (1,6329)	3.33	3.61	0.504	0.11
	Non-ADHD	3.4 (0.91), 6147		3.40	3.45		
Triglycerides, mmol/L	ADHD	2.27 (1.89), 202	4.7 (1,6675)	2.02	2.54	0.029	0.16
	Non-ADHD	2.03 (1.53), 6475		2.00	2.07		
**Outcome**		***n* (%) yes**	**χ^2^ (d.f.)**				**OR (95% CI)**
Current smoker	ADHD	232 (38.4%)	12.0 (1)	–	–	0.001	1.60 (1.22–2.1)
	Non-ADHD	7519 (28%)					

BMI, body mass index; SBP, systolic blood pressure; DBP, diastolic blood pressure; LDL, low-density lipoprotein.

Childhood ADHD problems were associated with BMI (*B* = 0.92 kg/m^2^, s.d. = 0.27–1.56), systolic (3.5 mmHg, s.d. = 1.4–5.6) and diastolic (2.2 mmHg, s.d. = 0.8–3.6) blood pressure, triglyceride levels (0.24 mmol/l, s.d. = 0.02–0.46) and being a current smoker (odds ratio OR = 1.6, s.d. = 1.2–2.1) but not with LDL cholesterol levels.

In separate analyses childhood ADHD was not associated with BMI at age 16 (ADHD group: mean 20.6 kg/m^2^; non-ADHD group: mean also 20.6 kg/m^2^) nor with BMI at age 11 (ADHD group: mean 17.3 kg/m^2^; non-ADHD group: mean 17.5 kg/m^2^).

Using current UK cut-offs for clinical action for mid-life hypertension, overweight, obesity and high LDL and high triglycerides (these vary across countries), odds ratios comparing the ADHD with the non-ADHD group were calculated (Table 2). Rates of those who met these cut-offs were higher in the ADHD group (OR > 1) but differences were robust only for obesity.

**Table 2:** Comparison of individuals with and without childhood attention-deficit hyperactivity disorder (ADHD) problems using UK-recommended cardiovascular risk factors thresholds for intervention

Outcome (cut-off), *n*	ADHD group, % (*n*)	Non-ADHD group, % (*n*)	Odds ratio (OR)	95% CI for OR	
				Upper	Lower
Hypertension (blood pressure ≥140/90 mmHg), 7894	13.7 (233)	11.3 (7661)	1.25	0.86	1.83
Overweight (BMI ≥25 kg/m^2^), 7873	65.4 (234)	65.4 (7639)	1.0	0.77	1.31
Obese (BMI ≥30 kg/m^2^), 7873	30.3 (234)	24.2 (7639)	1.36	1.03	1.81
Elevated triglycerides (≥1.7 mmol/L^a^), 6677	53.0 (202)	49.5 (6475)	1.15	0.87	1.52
Elevated triglycerides (≥2.3 mmol/L^b^), 6677	35.1 (202)	30.9 (6475)	1.21	0.90	1.62

a. Recommended fasting level (National Institute for Health and Care Excellence).

b. Recommended non-fasting level (HEART UK).

### Sensitivity analyses

As expected, sex was associated both with ADHD and systolic blood pressure in this sample (Supplementary eTable 1). Including sex as a covariate in the regression analysis of ADHD exposure and systolic blood pressure outcome, the association between ADHD and systolic blood pressure was attenuated. The interaction term between sex and ADHD did not significantly predict systolic blood pressure. Similarly, SES at birth attenuated the association between ADHD and systolic blood pressure (Supplementary eTable 2) and the interaction term did not achieve conventional levels of statistical significance.

### Multiple imputation

We imputed missing data for the 8016 individuals in our core group (Fig. 1). The patterns of missing data are shown in Supplementary eFigs 1 and 2; bivariate analyses with childhood ADHD as exposure and individual cardiovascular risk factors as outcomes using original and imputed data are shown in Supplementary eTable 4. Results for imputed data were similar to those found using the original data-set.

## Discussion

We found that individuals with childhood ADHD problems (‘squirmy, fidgety’ and ‘hardly ever still’) showed higher levels of multiple cardiovascular risk factors by age 44. These included higher blood pressure (both systolic and diastolic), triglyceride levels and BMI; they were also more likely to be current cigarette smokers. Similar results were obtained using the imputed data-set. When outcomes were examined categorically, to help inform clinicians as to how worthwhile cardiovascular risk profiling in psychiatry might be, out of 100 people with broadly defined ADHD aged 44–45, 14 in our sample met the UK threshold for treating hypertension and 33 for elevated triglycerides.

When we defined cardiovascular risk factors categorically, using current UK cut-offs for clinical intervention, we observed a higher odds ratio for each risk factor in those with childhood ADHD compared with those without. However, apart from obesity, we could not reject the null hypothesis; that is, the possibility that there is no difference in cardiovascular risk categories. The threshold for treating different cardiovascular risk factors (e.g. with antihypertensive medication, statins) varies across countries and is, for example, lower in the USA.

Given that adult psychiatrists are likely to be following up those with persistent ADHD rather than broadly defined childhood ADHD, these observations are probably an underestimate and now need to be examined in clinical adult psychiatry settings.

### Associations with BMI and cigarette smoking

There is a well-established association between ADHD and obesity (obesity defined as BMI ≥30 kg/m^2^).^16^ Genetic overlaps between ADHD and BMI have also been well documented^18^ and a recent study using two-sample Mendelian randomisation suggests, using genetic variants, a bidirectional causal relationship between ADHD and childhood obesity.^7^ We found a robust association between childhood ADHD problems and obesity but not between ADHD problems and overweight status. Interestingly, we only found elevated BMI in mid-life, not in childhood and adolescence. This suggests that adult psychiatrists need to monitor obesity status in particular.

Childhood ADHD was also associated with cigarette smoking in mid-life. Again, this link between smoking and ADHD is well established, with evidence suggesting that associations over the longer term may arise because of nicotine dependence linked to self-medication^19^ and increased likelihood of experiencing nicotine withdrawal effects such as irritability and difficulty concentrating on stopping.^20^ Genetic studies also have found evidence for genetic overlap between substance use disorder (including cigarette smoking) and ADHD.^21^ This is the first study to report mid-life smoking outcomes for individuals with ADHD symptoms assessed in childhood.

### Associations with blood pressure

Hypertension is the most important risk factor for coronary artery disease^8^ and the risk seems most pronounced for elevated systolic blood pressure.^15^ To our knowledge, no prospective studies have followed up children with ADHD or ADHD symptoms to examine blood pressure in mid-life. As medication used to treat ADHD has the propensity to cause elevated blood pressure, several studies have followed up children with ADHD on medication into early adulthood to check whether blood pressure becomes elevated; their findings are mixed, but a recent review has concluded that there is evidence for a slight elevation (3–8 mmHg) in systolic blood pressure.^22^ The individuals in our study were born in 1958. Medication for childhood ADHD was first approved in the 1960s but UK prescribing was relatively low until around 2000.^23^ Most individuals in our study would therefore not have been prescribed any medication for ADHD during childhood and adolescence. It is intriguing that, given our study findings, probably untreated childhood ADHD is also associated with a modest elevation of blood pressure in mid-life (age 44). Our study further elaborates recent studies on younger populations which found that untreated ADHD is associated with low blood pressure in late adolescence^24^ but that this finding does not persist into young adulthood.^25^ This would suggest the need to examine mechanisms that lead to the increase in blood pressure through adulthood for individuals with ADHD and the need for caution in interpreting any findings from more recent cohorts that focus on examining the long-term effects of medication for ADHD on blood pressure.

### Associations with cardiovascular risk and disease

Although there is growing recognition of the importance of cardiovascular disease and CVD risk factors in those with psychiatric disorders, especially depression, neurodevelopmental disorders such as ADHD that typically first manifest in childhood have often been ignored. Recent large-scale patient registry and clinical studies have suggested that clinically diagnosed ADHD is associated with cardiovascular diseases, but we do not know whether this is because of obesity and cigarette smoking alone. Our prospective study suggests that childhood ADHD problems in the general population are associated with multiple cardiovascular risk factors in mid-life. The findings from this study when taken together with clinical and registry-based studies highlight the importance of monitoring cardiovascular risk profiles in those with ADHD to prevent later CVD. Few psychiatrists will be trained in using CVD risk calculators and primary care physicians may not recognise people with ADHD as benefitting from repeated physical health checks.

### Systolic blood pressure, sex and SES

ADHD and systolic blood pressure are known to be associated with both sex and socioeconomic status. Sensitivity checks were conducted to examine males and females separately and higher and lower SES at birth. These suggested that the relationship between ADHD and systolic blood pressure is attenuated by sex; we observed the same attenuation for SES at birth. We did not detect robust evidence that the association between ADHD and systolic blood varied by sex or SES at birth. However, given the small sample size, there is a need for caution in interpreting these results, especially given robust evidence of elevated cardiovascular disease rates in those with ADHD in larger patient registry data, regardless of sex and SES.^26^

### Strengths and limitations

Although this study benefits from using a population-based birth cohort followed up until mid-life with repeated direct assessments there are some limitations. In common with most population cohorts, there is some loss to follow-up, which leads to some missing data. However, results using multiple imputation were very similar to those for the primary analysis, although data on which to base imputations were limited, as were detailed data on medications and healthcare use. Another limitation was that ADHD assessment during childhood in the 1960s was limited to the Rutter Scale. The NCDS includes only two questions, with a focus more on the hyperactive rather than the inattentive domain – a decision that might have seemed appropriate at that time. However, both parent and teacher ratings were available and used for the categorisation, the questionnaires have been validated against diagnoses and our prevalence rate of broadly defined ADHD (3%) is not dissimilar to rates observed using diagnostic interviews. It is now known that childhood ADHD symptoms and impairment persist in most individuals into adult life and that adult ADHD requires clinical attention.^27^ However, the NCDS like many other population cohorts and volunteer cohorts, such as UK Biobank, has never included any measures of adult ADHD. Other limitations are that until information from this cohort at an older age is available, it is not possible to replicate recent health registry findings on ADHD diagnosis and CVD associations. However, the registry studies are unable to directly examine CVD risk factors, which we were able to do. Moreover, triangulation of findings on CVD and CVD risk from patient-based registry studies, genetic studies^28^ and now a population cohort study provides greater confidence in conclusions across these different designs.

### Implications

As noted, childhood ADHD problems are associated with higher levels of multiple risk factors in mid-life that are particularly associated with the development of CVD.^28^ However, individuals with ADHD may be less likely to consult a primary care physician about their physical health and may therefore be less likely to have the opportunity to discuss the benefits of preventive strategies, including lifestyle measures and interventions such as treating hypertension and hyperlipidaemia. Also, monitoring physical health may not be a priority for adult mental health services. For example, guidance on managing ADHD from the UK's National Institute for Health and Care Excellence does not mention the need for physical health screening in adulthood unless related to medication.^29^ This study adds to the literature in highlighting the importance of considering physical and mental health together with neurodevelopmental disorders. Taking all the findings across different studies together, CVD risk monitoring and early prevention of CVD should be part of follow-up for individuals with a history of ADHD, given that CVD risk factors are amenable to intervention.

## Data Availability

The data that supports the findings of this study are available from the UK Data Service-National Child Development Study (https://ukdataservice.ac.uk/help/datatypes/longitudinal-data-studies/). Restrictions apply to the availability of these data, which were used under licence for this study.

## Supplementary material

For supplementary material accompanying this paper visit https://doi.org/10.1192/bjp.2023.90. click here to view supplementary material

## References

[1] Polanczyk Gv, Salum GA, Sugaya LS, Caye A, Rohde LA. Annual research review: a meta-analysis of the worldwide prevalence of mental disorders in children and adolescents. J Child Psychol Psychiatry 2015; 56: 345–65. 25649325 10.1111/jcpp.12381

[2] Fayyad J, Sampson NA, Hwang I, Adamowski T, Aguilar-Gaxiola S, Al-Hamzawi A, et al. The descriptive epidemiology of DSM-IV adult ADHD in the World Health Organization World Mental Health Surveys. Atten Defic Hyperact Disord 2017; 9: 47–65. 27866355 10.1007/s12402-016-0208-3 PMC5325787

[3] Dalsgaard S, Ostergaard SD, Leckman JF, Mortensen PB, Pedersen MG. Mortality in children, adolescents, and adults with attention deficit hyperactivity disorder: a nationwide cohort study. Lancet 2015; 385: 2190–6. 25726514 10.1016/S0140-6736(14)61684-6

[4] London AS, Landes SD. Attention deficit hyperactivity disorder and adult mortality. Prev Med 2016; 90: 8–10. 27343403 10.1016/j.ypmed.2016.06.021

[5] Catalá-López F, Hutton B, Page MJ, Driver JA, Ridao M, Alonso-Arroyo A, et al. Mortality in persons with autism spectrum disorder or attention-deficit/hyperactivity disorder: a systematic review and meta-analysis. JAMA Pediatr 2022; 176(4): e216401. 35157020 10.1001/jamapediatrics.2021.6401 PMC8845021

[6] Li L, Chang Z, Sun J, Garcia-Argibay M, Du Rietz E, Dobrosavljevic M, et al. Attention-deficit/hyperactivity disorder as a risk factor for cardiovascular diseases: a nationwide population-based cohort study. World Psychiatry 2022; 21: 452–9. 36073682 10.1002/wps.21020 PMC9453905

[7] Leppert B, Riglin L, Wootton RE, Dardani C, Thapar A, Staley JR, et al. The effect of attention deficit/hyperactivity disorder on physical health outcomes: a 2-sample Mendelian randomization study. Am J Epidemiol 2021; 190:1047–55. 10.1093/aje/kwaa273 PMC8168225 33324987

[8] Yusuf S, Joseph P, Rangarajan S, Islam S, Mente A, Hystad P, et al. Modifiable risk factors, cardiovascular disease, and mortality in 155 722 individuals from 21 high-income, middle-income, and low-income countries (PURE): a prospective cohort study. Lancet 2020; 395: 795–808. 31492503 10.1016/S0140-6736(19)32008-2 PMC8006904

[9] Power C, Elliott J. Cohort profile: 1958 British birth cohort (National Child Development Study). Int J Epidemiol 2006; 35: 34–41. 16155052 10.1093/ije/dyi183

[10] Thapar A, Cooper M. Attention deficit hyperactivity disorder. Lancet 2016; 387: 1240–50. 26386541 10.1016/S0140-6736(15)00238-X

[11] National Child Development Study. Ethical Review and Consent. Centre for Longitudinal Studies, Institute of Education, University of London, 2014 (https://cls.ucl.ac.uk/wp-content/uploads/2017/07/NCDS-Ethical-review-and-Consent-2014.pdf [accessed 23 Jun 2023]).

[12] Rutter M, Tizard J, Whitmore K. Education, Health and Behavior. Longman Group, 1970.

[13] Stott DH, Sykes EG. The social adjustment of children: the Bristol Social Adjustment Guides. AMA Arch Gen Psychiatry 1959; 1(5): 556.

[14] Collishaw S. Annual research review: secular trends in child and adolescent mental health. J Child Psychol Psychiatry 2015; 56(3): 370–93. 25496340 10.1111/jcpp.12372

[15] Kannel WB. Elevated systolic blood pressure as a cardiovascular risk factor. Am J Cardiol 2000; 85: 251–5. 10955386 10.1016/s0002-9149(99)00635-9

[16] Cortese S, Moreira-Maia CR, St Fleur D, Morcillo-Peñalver C, Rohde LA, Faraone Sv. Association between ADHD and obesity: a systematic review and meta-analysis. Am J Psychiatry 2016; 173: 34–43. 26315982 10.1176/appi.ajp.2015.15020266

[17] Silverwood R, Narayanan M, Dodgeon B, Ploubidis G. Handling Missing Data in the National Child Development Study: User Guide (Version 2). UCL Centre for Longitudinal Studies, 2021.

[18] Peters T, Nüllig L, Antel J, Naaresh R, Laabs BH, Tegeler L, et al. The role of genetic variation of BMI, body composition, and fat distribution for mental traits and disorders: a look-up and mendelian randomization study. Front Genet 2020; 11: 373. 10.3389/fgene.2020.00373 PMC7186862 32373164

[19] van Amsterdam J, van der Velde B, Schulte M, van den Brink W. Causal factors of increased smoking in ADHD: a systematic review. Subst Use Misuse 2018; 53: 432–45. 29039714 10.1080/10826084.2017.1334066

[20] Pomerleau CS, Downey KK, Snedecor SM, Mehringer AM, Marks JL, Pomerleau OF. Smoking patterns and abstinence effects in smokers with no ADHD, childhood ADHD, and adult ADHD symptomatology. Addict Behav 2003; 28: 1149–57. 12834657 10.1016/s0306-4603(02)00223-x

[21] Vilar-Ribó L, Sánchez-Mora C, Rovira P, Richarte V, Corrales M, Fadeuilhe C, et al. Genetic overlap and causality between substance use disorder and attention-deficit and hyperactivity disorder. Am J Med Gen Part B 2021; 186: 140–50. 10.1002/ajmg.b.32827 33244849

[22] Fay TB, Alpert MA. Cardiovascular effects of drugs used to treat attention-deficit/hyperactivity disorder. Part 2: Impact on cardiovascular events and recommendations for evaluation and monitoring. Cardiol Rev 2019; 27: 173–8. 30531411 10.1097/CRD.0000000000000234

[23] Renoux C, Shin JY, Dell'Aniello S, Fergusson E, Suissa S. Prescribing trends of attention-deficit hyperactivity disorder (ADHD) medications in UK primary care, 1995–2015. Br J Clin Pharmacol 2016: 858–68. 27145886 10.1111/bcp.13000 PMC5338115

[24] Garcia-Argibay M, du Rietz E, Hartman CA, Lichtenstein P, Chang Z, Larsson H. Cardiovascular risk factors in attention-deficit/hyperactivity disorder: a family design study of Swedish conscripts. 2022; 31(4): e1930. 10.1002/mpr.1930 PMC9720218 35765813

[25] Schulz J, Huber F, Schlack R, Hölling H, Ravens-Sieberer U, Meyer T, et al. The association between low blood pressure and attention-deficit hyperactivity disorder (ADHD) observed in children/adolescents does not persist into young adulthood: a population-based ten-year follow-up study. Int J Environ Res Public Health 2021; 18: 1864. 33672943 10.3390/ijerph18041864 PMC7918102

[26] Bowerman BL, O'Connell RT. Linear Statistical Models: An Integrated Approach. Duxbury Press, 1990.

[27] Caye A, Swanson J, Thapar A, Sibley M, Arseneault L, Hechtman L, et al. Life span studies of ADHD-conceptual challenges and predictors of persistence and outcome. Curr Psychiatry Rep 2016; 18(12): 111. 10.1007/s11920-016-0750-x PMC5919196 27783340

[28] Wilson PWF. Overview of Established Risk Factors for Cardiovascular Disease. UpToDate, 2023 (https://www.uptodate.com/contents/overview-of-established-risk-factors-for-cardiovascular-disease [accessed 21 Feb 2023]).

[29] National Institute for Health and Care Excellence. Recommendations. In Attention deficit Hyperactivity Disorder: Diagnosis and Management (NICE Guideline NG87). NICE (https://www.nice.org.uk/guidance/ng87/chapter/recommendations [accessed 9 Mar 2022]).

