# Supplementary material for: Childhood attention-deficit hyperactivity disorder problems and mid-life cardiovascular risk: prospective population cohort study
Source: Br J Psychiatry. Author manuscript; Available in PMC 2024 Jan 11. (PMC7615511; doi:10.1192/bjp.2023.90)
Supplement: Supplementary file [file EMS192899-supplement-Supplementary_file.docx]

**Supplement: Childhood Attention Deficit Hyperactivity Disorder problems and mid-life cardiovascular risk: a prospective population cohort study. Thapar et.al**

**Sex: Association with ADHD and SBP**

ADHD(n=239): Males (63.2%) Females (36.8%)

ADHD association with being male (Odds Ratio 1.4; 95% CI 1.2-1.6)

Systolic blood pressure(mmHg): Males (mean 132.7, SD 14.8) Females (mean 120.2 SD 15.4)

| **Predictor Variable** | **Unstandardised coefficient** | | **T value** | **P value** | **95% CI for B** | | | **F test(Degrees of freedom)** |
| --- | --- | --- | --- | --- | --- | --- | --- | --- |
|  | **B** | **SE** |  |  | | **Lower** | **Upper** |  |
| ADHD | 1.1 | 1.6 | 0.65 | 0.52 | | -2.2 | 4.3 | 454.9(3,7890) |
| Sex of child | -12.5 | 0.35 | -36.2 | <0.001 | | -13.2 | -11.8 |  |
| Sex X ADHD interaction | 1.1 | 2.1 | 0.52 | 0.60 | | -3.0 | 5.2 |  |
| Constant | 145.3 | 0.5 | 264.5 | <0.001 | | 144.2 | 146.4 |  |

**eTable 1: Multiple regression including sex: ADHD (predictor) and SBP (outcome)**

**eTable 2: Multiple regression including SES: ADHD (predictor) and SBP (outcome)**

| **Predictor Variable** | **Unstandardised coefficient** | | **T value** | **P value** | **95% CI for B** | | | **F test (degrees of Freedom)** |
| --- | --- | --- | --- | --- | --- | --- | --- | --- |
|  | **B** | **SE** |  |  | | **Lower** | **Upper** |  |
| ADHD | 2.5 | 2.0 | 1.21 | 0.22 | | -1.51 | 6.5 | 5.70 (3,7890) |
| SES group | -1.2 | 0.46 | -2.6 | 0.009 | | -2.1 | -0.29 |  |
| SES X ADHD interaction | 1.29 | 2.41 | 0.53 | 0.59 | | -3.4 | 6.0 |  |
| Constant | 127.3 | 0.41 | 310.6 | <0.001 | | 126.6 | 128.2 |  |

**Multiple imputation**

Multiple imputation was recommended as the method of choice for missing variables in NCDS (Silverwood, R., Narayanan, M., Dodgeon, B., Ploubidis, G. (2021) Handling Missing Data in the National Child Development Study: User Guide (Version 2). London: UCL Centre for Longitudinal Studies).

The same document suggested list of auxiliary variables to use if variables were missing at different age points. For our purposes we used as many of the auxiliary variables that we have access to, and that were recommended for missing data at the age 44/45 biomedical survey (Social problems in the household at 7, mathematics comprehension at age 11, number of persons per room, emotional/behaviour problems at age 16, Social class of mother’s husband and father, conduct problems, how long since child drank alcohol, dad staying on in school after minimum age and school attendance).

We used auxiliary variables collected at birth, childhood and during adolescence. We also included key interaction terms (sex with ADHD), any earlier measures of mid-life CV outcomes (BMI and age 7 and BMI at age 11) and some key other variables (lung function (Forced expiratory function) and deaths from ages 7 to 58 years). These variables (except for the sex*ADHD interaction term) are summarised below in eTable3.

We initially examined the patterns of missingness. The results are displayed below in eFigure 1 & eFigure 2. This procedure was implemented using SPSS 27.

We tested whether included variables were significant predictors of missingness. We dropped BMI at age 7, BMI at age 11 and cognitive ability summary at age 7 which did not predict missingness. We then carried out a multiple imputation to minimise the effect of missing data for the 8016 individuals who were the index group for the core analysis. We specified a multivariate normal model with a random seed (53421). We chose 10 imputations as this ensured efficiency of point estimates but also because stability of standard error estimates was not improved with higher numbers of imputations. These procedures were implemented using STATA version16.1.

| *Variable description-Brown:birth; Green age 7; Blue age 11; Purple age 16* |  | *N* | *Mean* | *SD* | *Missing* | |
| --- | --- | --- | --- | --- | --- | --- |
|  | *Role in imputation* |  |  |  | *N* | *Percent* |
|  |  |  |  |  |  |  |
| **Forced expiratory volume** | Impute and Predictor | 9090 | 3.25 | 0.87 | 9468 | 51.0 |
| **Depression summary score at age 44/45** | Impute and Predictor | 9296 | 0.29 | 0.73 | 9262 | 49.9 |
| **Serum triglycerides** | Impute and Predictor | 7799 | 2.05 | 1.57 | 10759 | 58.0 |
| **Serum LDL cholesterol** | Impute and Predictor | 7391 | 3.42 | 0.91 | 11167 | 60.2 |
| **Mean Systolic blood pressure** | Impute and Predictor | 9230 | 126.5 | 16.4 | 9328 | 50.3 |
| **Mean Diastolic blood pressure** | Impute and Predictor | 9229 | 78.7 | 10.7 | 9329 | 50.3 |
| **Whether or not current smoker** | Impute and Predictor | 9069 |  |  | 9489 | 51.1 |
| **Body Mass Index at age 44 years** | Impute and Predictor | 9209 | 27.39 | 4.96 | 9349 | 50.4 |
| **Body mass Index at 16** | Impute and Predictor | 11039 | 20.61 | 2.86 | 7519 | 40.5 |
| **Body mass Index at 11** | Dropped^1^ | 12498 | 17.46 | 2.56 | 6060 | 32.7 |
| **Body mass Index at 7** | Dropped^1^ | 13442 | 15.93 | 1.815 | 5116 | 27.6 |
| **Cognitive ability summary at 11** | Predictor | 14131 | 42.94 | 16.14 | 4427 | 23.9 |
| **Cognitive ability summary at 7** | Dropped ^1^ | 14999 | -0.03 | 1.89 | 3559 | 19.2 |
| **Social problems household at 7** | Predictor | 10994 | 0.36 | .90 | 7564 | 40.8 |
| **Test 2 Mathematics comprehension at 11** | Predictor | 11920 | 12.75 | 7.00 | 6638 | 35.8 |
| **Death status by age 58** | Predictor | 17733 |  |  | 825 | 4.4 |
| **Social class at birth dichotomised** | Predictor | 9376 |  |  | 9182 | 49.5 |
| **ADHD at 7-all those with valid data** | Predictor | 14185 |  |  | 4373 | 23.6 |
| **ADHD at 7-also participated in biomedical sweep** | Predictor | 8016 |  |  | 10542 | 56.8 |
| **Sex** | Predictor | 18554 |  |  | 4 | .0 |
| **No. of persons per room** | Predictor | 16920 |  |  | 1638 | 8.8 |
| **Emotional or behavioural problem at 16** | Predictor | 11366 |  |  | 7192 | 38.8 |
| **Social class of mother’s husband** | Predictor | 16458 |  |  | 2100 | 11.3 |
| **Social class of mother’s father when she left school** | Predictor | 14289 |  |  | 4269 | 23.0 |
| **Conduct problems** | Predictor | 9751 |  |  | 8807 | 47.5 |
| **How long since child drank alcohol** | Predictor | 12006 |  |  | 6552 | 35.3 |
| **Dad stayed on in school after minimum age** | Predictor | 14051 |  |  | 4507 | 24.3 |
| **School Attendance** | Predictor | 14322 |  |  | 4236 | 22.8 |
| Abnormality during pregnancy | Predictor | 17404 |  |  | 1154 | 6.2 |

**eTable 3: Missing value analysis and role of variables in multiple imputation**

^1^ not significant predictor of missingness of key outcome variables.

Percent=percentage

**eFigure 1: Missing data pattern illustration (not all variables included because of constraints of the graphical tool)**

**
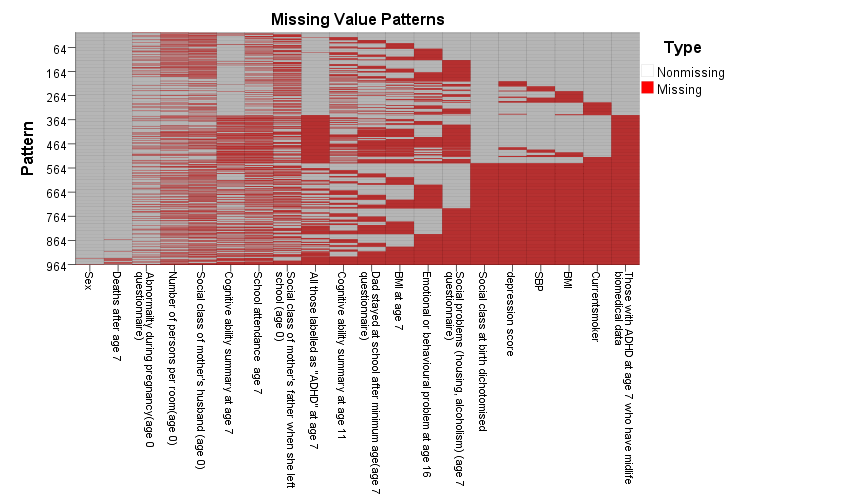
**

**Y axis: pattern number for missing (red) and non-missing (grey) data with higher values (bottom of Y axis) showing more missing data (note not possible to show every single pattern which is why the Y axis starts at 964-the pattern with the most missing data).**

**eFigure 2: Frequency of missing data patterns (percentsum= percentage of cases)**

**
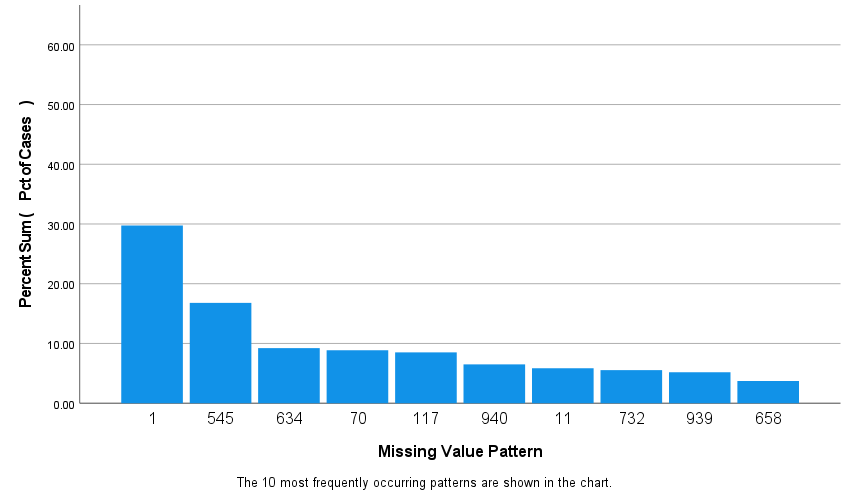
**

**eTable 4: Associations between childhood ADHD and mid-life cardiovascular risk factors using imputed data**

| **Outcome** | **Regression coefficient (B)** | **95%CI B** | **P value** |
| --- | --- | --- | --- |
| Mid-life BMI (Kg/m^2)^ | | | |
| Original data (n=7873) | 0.92 | 0.27-1.56 | 0.005 |
| Imputed data (n=8016) | 0.83 | 0.18-1.48 | 0.012 |
| SBP (mmHg) | | | |
| Original data (n=7894) | 3.5 | 1.36-5.63 | 0.001 |
| Imputed data(n=8016) | 3.6 | 1.43-5.70 | 0.001 |
| DBP (mmHg) | | | |
| Original data (n=7894) | 2.2 | 0.81-3.61 | 0.002 |
| Imputed data(n=8016) | 2.2 | 0.85-3.64 | 0.002 |
| TG (mmol/l) | | | |
| Original data (n=6677) | 0.24 | 0.02-0.45 | 0.03 |
| Imputed data(n=8016) | 0.24 | 0.03-0.46 | 0.02 |
| LDL cholesterol (mmol/l) | | | |
| Original data (n=6331) | 0.05 | -0.09-0.18 | 0.50 |
| Imputed data(n=8016) | 0.06 | -0.08-0.209 | 0.40 |
| Current Smoking | OR | 95% CI OR | P-value |
| Original data (n=7751) | 1.60 | 1.22-2.10 | 0.001 |
| Imputed data (n=8016) | 1.29 | 1.18-1.40 | 0.000 |
